# A new species of the Asian leaf litter toad genus *Leptobrachella* Smith, 1925 (Anura, Megophryidae) from northwest Guizhou Province, China

**DOI:** 10.3897/zookeys.1021.60729

**Published:** 2021-03-02

**Authors:** Yan-Lin Cheng, Sheng-Chao Shi, Jiaqi Li, Jing Liu, Shi-Ze Li, Bin Wang

**Affiliations:** 1 Department of Resources and Environment, Moutai Institute, Renhuai 564500, China Moutai Institute Renhuai China; 2 Chengdu Institute of Biology, Chinese Academy of Sciences, Chengdu 610041, China Chengdu Institute of Biology, Chinese Academy of Sciences Chengdu China; 3 Nanjing Institute of Environmental Sciences, Ministry of Ecology and Environment of China, Nanjing 210042, China Ministry of Ecology and Environment of China Nanjing China

**Keywords:** *Leptobrachella jinshaensis* sp. nov., molecular phylogenetic analyses, morphology, Taxonomy

## Abstract

A new species of the Asian leaf litter toad genus *Leptobrachella* is described from Guizhou Province, China. Molecular phylogenetic analyses support the new species as an independent lineage deeply nested in the *Leptobrachella* clade. The new species is distinguished from its congeners by a combination of the following morphological characters: body size medium (SVL 29.7–31.2 mm in five adult males); dorsal skin shagreened, some of the granules forming longitudinal short skin ridges; tympanum distinctly discernible, slightly concave; supra-axillary, femoral, pectoral and ventrolateral glands distinctly visible; absence of webbing and lateral fringes on fingers; toes with narrow lateral fringes but without webbing; heels overlapping when thighs are positioned at right angles to the body; tibia-tarsal articulation reaching the middle of eye when leg stretched forward. The discovery highlighted the underestimated species diversity in the *Leptobrachella* toads in southwestern China.

## Introduction

The Asian leaf litter toads of the genus *Leptobrachella* Smith, 1925 (Anura, Megophryidae) are widely distributed from southern China west to northeastern India and Myanmar, through mainland Indochina to peninsular Malaysia and the island of Borneo (Frost 2020). Many species in this genus have been classified into *Leptolalax* Dubois, 1983 (e.g., Fei et al. 2009, 2012), but Chen et al. (2018) placed *Leptolalax* as a junior synonym of *Leptobrachella* based on large-scale molecular analyses. Currently, the genus *Leptobrachella* contains 82 species (Frost 2020) but a series of cryptic species is still suggested by molecular phylogenetic analyses (Chen et al. 2018). To date, 25 species of this genus have been recorded in China, i.e., *L. alpina* (Fei, Ye & Li, 1990) and *L. bourreti* (Dubois, 1983) from Yunnan and Guangxi; *L. eos* (Ohler, Wollenberg, Grosjean, Hendrix, Vences, Ziegler & Dubois, 2011), *L. nyx* (Ohler, Wollenberg, Grosjean, Hendrix, Vences, Ziegler & Dubois, 2011), *L. pelodytoides* (Boulenger, 1893), *L. tengchongensis* (Yang, Wang, Chen & Rao, 2016), *L. yingjiangensis* (Yang, Zeng & Wang, 2018), *L. feii* (Chen, Yuan & Che, 2020), *L. flaviglandulosa* (Chen, Wang & Che, 2020), and *L. niveimontis* (Chen, Poyarkov, Yuan & Che, 2020) from Yunnan; *L. laui* (Sung, Yang & Wang, 2014) and *L. yunkaiensis* Wang, Li, Lyu & Wang, 2018 from Guangdong and Hong Kong; *L. liui* (Fei & Ye, 1990) from Fujian, Jiangxi, Guangdong, Guangxi, Hunan, and Guizhou; *L. oshanensis* (Liu, 1950) from Gansu, Sichuan, Chongqing, Guizhou, and Hubei; *L. purpuraventra* (Wang, Li, Li, Chen & Wang, 2019), *L. bijie* (Wang, Li, Li, Chen & Wang, 2019), *L. chishuiensis* (Li, Liu, Wei & Wang, 2020), and *L. suiyangensis* (Luo, Xiao, Gao & Zhou, 2020) from Guizhou; *L. purpurus* (Yang, Zeng & Wang, 2018), *L. ventripunctata* (Fei, Ye & Li, 1990) from Guizhou and Yunnan; *L. mangshanensis* (Hou, Zhang, Hu, Li, Shi, Chen, Mo & Wang, 2018) from Hunan; and *L. sungi* (Lathrop, Murphy, Orlov & Ho, 1998), *L. maoershanensis* (Yuan, Sun, Chen, Rowley & Che, 2017), *L. shangsiensis* (Chen, Liao, Zhou & Mo, 2019), and *L. wuhuangmontis* (Wang, Yang & Wang, 2018) from Guangxi. Among them, ten *Leptobrachella* species occur in Guizhou Province, China, highlighting the high species diversity of the genus in this region.

In recent years, we collected some specimens of *Leptobrachella* from northwest Guizhou Province, China. Molecular phylogenetic analyses, morphological comparisons, and bioacoustics data consistently indicated these specimens as an undescribed species of *Leptobrachella*. We describe it herein as a new species.

## Materials and methods

### Specimens

**Figure 1.**
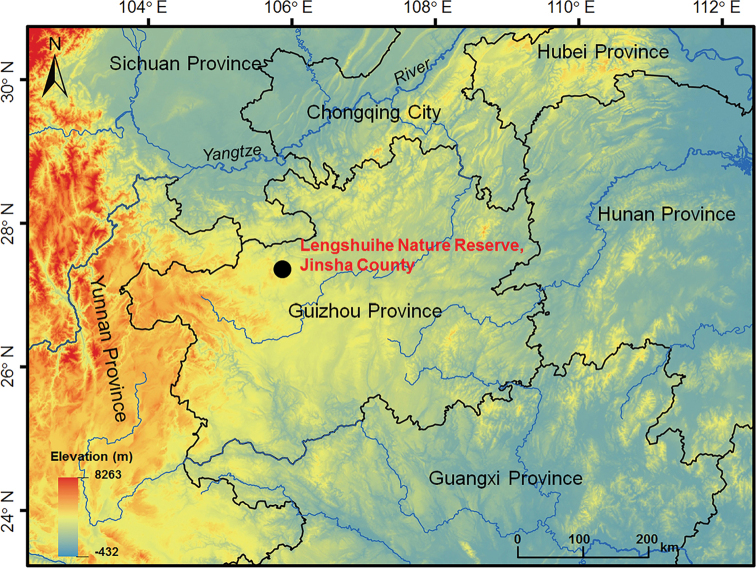
Location of the type locality of *Leptobrachella jinshaensis* sp. nov., Lengshuihe Nature Reserve, Jinsha County, Guizhou Province, China.

Five adult males of the new species were collected on 16 May 2020 from Lengshuihe Nature Reserve, Jinsha County, Guizhou Province, China (Fig. 1; Table 1). After taking photographs, toads were euthanised using isoflurane, and then the specimens were fixed in 10% buffered formalin. Tissue samples were taken and preserved separately in 95% ethanol prior to fixation. Specimens were deposited in Chengdu Institute of Biology, Chinese Academy of Sciences (**CIB**, **CAS**).

**Table 1.** Information for samples used in molecular phylogenetic analyses in this study.

ID	Species	Voucher	Locality	GenBank accession number
1	*Leptobrachella jinshaensis* sp. nov.	CIBJS20200516001	Lengshuihe Nature Reserve, Jinsha County, Guizhou Province, China	MT814014
2	*Leptobrachella jinshaensis* sp. nov.	CIBJS20200516002	Lengshuihe Nature Reserve, Jinsha County, Guizhou Province, China	MT814015
3	*Leptobrachella jinshaensis* sp. nov.	CIBJS20200516003	Lengshuihe Nature Reserve, Jinsha County, Guizhou Province, China	MT814016
4	*Leptobrachella jinshaensis* sp. nov.	CIBJS20200516004	Lengshuihe Nature Reserve, Jinsha County, Guizhou Province, China	MT814017
5	*Leptobrachella jinshaensis* sp. nov.	CIBJS20200516005	Lengshuihe Nature Reserve, Jinsha County, Guizhou Province, China	MT814018
6	*Leptobrachella chishuiensis*	CIBCS20190518047	Alsophila National Nature Reserve, Chishui City, Guizhou Province, China	MT117053
7	*Leptobrachella chishuiensis*	CIBCS20190518042	Alsophila National Nature Reserve, Chishui City, Guizhou Province, China	MT117054
8	*Leptobrachella chishuiensis*	CIBCS20190518043	Alsophila National Nature Reserve, Chishui City, Guizhou Province, China	MT117055
9	*Leptobrachella bijie*	SYS a007313/CIB110002	Mt. Zhaozi Nature Reserve, Bijie City, Guizhou Province, China	MK414532
10	*Leptobrachella bijie*	SYS a007314	Mt. Zhaozi Nature Reserve, Bijie City, Guizhou Province, China	MK414533
11	*Leptobrachella bijie*	SYS a007315	Mt. Zhaozi Nature Reserve, Bijie City, Guizhou Province, China	MK414534
12	*Leptobrachella suiyangensis*	GZNU20180606002	Huoqiuba Nature Reserve, Suiyang County, Guizhou, China	MK829648
13	*Leptobrachella suiyangensis*	GZNU20180606006	Huoqiuba Nature Reserve, Suiyang County, Guizhou, China	MK829649
14	*Leptobrachella suiyangensis*	GZNU20180606005	Huoqiuba Nature Reserve, Suiyang County, Guizhou, China	MK829650
15	*Leptobrachella niveimontis*	KIZ015744	Daxueshan Nature Reserve, Yunnan, China	MH055878
16	*Leptobrachella purpuraventra*	SYS a007081	Wujing Nature Reserve, Bijie City, Guizhou Province, China	MK414517
17	*Leptobrachella purpuraventra*	SYS a007277/CIB110003	Wujing Nature Reserve, Bijie City, Guizhou Province, China	MK414518
18	*Leptobrachella purpuraventra*	SYS a007278	Wujing Nature Reserve, Bijie City, Guizhou Province, China	MK414519
19	*Leptobrachella bourreti*	AMS R 177673	Lao Cai Province, Vietnam	KR018124
20	*Leptobrachella purpurus*	SYS a006530	Yingjiang County, Yunnan Province, China	MG520354
21	*Leptobrachella alpina*	KIZ046816	Huangcaoling, Yunnan Province, China	MH055866
22	*Leptobrachella oshanensis*	KIZ025776	Emei Shan, Emei Shan City, Sichuan Province, China	MH055895
23	*Leptobrachella eos*	MNHN:2004.0278	Phongsaly Province, Laos	JN848450
24	*Leptobrachella tengchongensis*	SYS a004598	Tengchong County, Yunnan Province, China	KU589209
25	*Leptobrachella puhoatensis*	AMS:R184852	Pu Hoat Nature Reserve, Nghe An Province, Vietnam	KY849588
26	*Leptobrachella namdongensis*	VNUF A.2017.37	Thanh Hoa Provincen, Vietnam	MK965389
27	*Leptobrachella petrops*	AMS:R184826	Vietnam	KY459997
28	*Leptobrachella khasiorum*	SDBDU 2009.329	East Khasi Hills, Meghalaya, India	KY022303
29	*Leptobrachella yingjiangensis*	SYS a006532	Yingjiang County, Yunnan Province, China	MG520351
30	*Leptobrachella mangshanensis*	MSZTC201701	Mt. Mang, Yizhang County, Hunan Province, China	MG132196
31	*Leptobrachella liui*	SYS a001597	Mt. Wuyi, Wuyishan City, Fujian Provnce, China	KM014547
32	*Leptobrachella laui*	SYS a001507	Mt. Wutong, Shenzhen City, Guangdong Province, China	KM014544
33	*Leptobrachella yunkaiensis*	SYS a004664 / CIB107272	Dawuling Forest Station, Maoming City, Guangdong Province, China	MH605585
34	*Leptobrachella maoershanensis*	KIZ019385	Mt. Maoer Nature Reserve, Ziyuan County, Guangxi Province, China	KY986930
35	*Leptobrachella flaviglandulosa*	KIZ016072	Xiaoqiaogou Nature Reserve, Yunnan, China	MH055934
36	*Leptobrachella zhangyapingi*	KIZ07258	Pang Num Poo, Chiang Mai Province, Thailand	MH055864
37	*Leptobrachella sungi*	ROM 20236	Tam Dao, Vinh Phuc, Vietnam	MH055858
38	*Leptobrachella isos*	VNMN A 2015.4/AMS R 176480	Gia Lai Province, Vietnam	KT824769
39	*Leptobrachella firthi*	AMS R 176524	Kon Tum Province, Vietnam	JQ739206
40	*Leptobrachella minimus*	KUHE:19201	Thailand	LC201981
41	*Leptobrachella ventripunctata*	SYS a004536	Zhushihe, Yunnan Province, China	MH055831
42	*Leptobrachella feii*	KIZ048893	Xiaoqiaogou Nature Reserve, Yunnan, China (E)	MH055841
43	*Leptobrachella aerea*	ZFMK 86362	Quang Binh Provice, Vietnam	JN848409
44	*Leptobrachella pluvialis*	MNHN:1999.5675	Mt. Fan Si Pan, Lao Cai Province, Vietnam	JN848391
45	*Leptobrachella shangsiensis*	NHMG1704003	Shangsi County, Guangxi Zhuang minority Autonomous Region, China	MK095463
46	*Leptobrachella wuhuangmontis*	SYS a003500 / CIB107274	Mt. Wuhuang, Pubei County, Guangxi Zhuang minority Autonomous Region, China	MH605581
47	*Leptobrachella nahangensis*	ROM 7035	Na Hang Nature Reserve, Tuyen Quang, Vietnam	MH055853
48	*Leptobrachella nyx*	AMNH A163810	Ha Giang Province, Vietnam	DQ283381
49	*Leptobrachella tuberosa*	ZMMU-NAP-02275	Kon Ka Kinh National Park, Gia Lai, Vietnam	MH055959
50	*Leptobrachella botsfordi*	VNMN 03682	Fansipan, Lao Cai, Vietnam	MH055953
51	*Leptobrachella pallida*	UNS00510	Lam Dong Province, Vietnam	KR018112
52	*Leptobrachella kalonensis*	IEBR A.2015.15	Binh Thuan Province, Vietnam	KR018114
53	*Leptobrachella bidoupensis*	NAP-01453	Lam Dong Province, Vietnam	KP017573
54	*Leptobrachella tadungensis*	UNS00515	Dak Nong Province, Vietnam	KR018121
55	*Leptobrachella maculosa*	AMS R 177660	Ninh Thuan Province, Vietnam	KR018119
56	*Leptobrachella pyrrhops*	ZMMU ABV-00148	Loc Bao, Lam Dong Provice, Vietnam	KP017575
57	*Leptobrachella macrops*	IEBR A.2017.9	Hon Den Mt., Phu Yen Province, Vietnam	MG787990
58	*Leptobrachella melica*	MVZ 258197	Virachey National Park, Ratanakiri Province, Cambodia	HM133599
59	*Leptobrachella applebyi*	AMS R171704	Song Thanh, Quang Nam, Vietnam	HM133598
60	*Leptobrachella rowleyae*	ITBCZ 2783	Son Tra, Da Nang City, Vietnam	MG682552
61	*Leptobrachella ardens*	AMS R 176463	Gia Lai Province, Vietnam	KR018110
62	*Leptobrachella crocea*	AMS R 173740	Kon Tum, Vietnam	MH055954
63	*Leptobrachella melanoleuca*	KUHE 23840	Thailand	LC201997
64	*Leptobrachella fuliginosa*	KUHE:20172	Thailand	LC201985
65	*Leptobrachella itiokai*	KUHE:55897	Mulu NP, Sarawak, Borneo, Malaysia	LC137805
66	*Leptobrachella brevicrus*	ZMH A09365	Sarawak: Gunung Mulu National Park: Small stream of the Sungei Tapin, Malaysia	KJ831302
67	*Leptobrachella parva*	KUHE 55308	Mulu NP, Sarawak, Borneo, Malaysia	LC056791
68	*Leptobrachella baluensis*	SP 21604	Tambunan, Sabah, Borneo, Malaysia	LC056792
69	*Leptobrachella mjobergi*	KUHE 17064	Gading NP, Sarawak, Borneo, Malaysia	LC056785
70	*Leptobrachella juliandringi*	SRC 00230/KUHE 49815	Mulu NP, Sarawak, Borneo, Malaysia	LC056779
71	*Leptobrachella arayai*	BORNEEISIS 22931	Liwagu, Kinabalu, Borneo, Malaysia	AB847558
72	*Leptobrachella hamidi*	KUHE 17545	Borneo, Malaysia	AB969286
73	*Leptobrachella marmorata*	KUHE 53227	Annah Rais, Padawan, Kuching Division, Sarawak, Malaysia	AB969289
74	*Leptobrachella maura*	SP 21450	Kinabalu, Sabah, Malaysia	AB847559
75	*Leptobrachella gracilis*	KUHE 55624	Camp 1, Gunung Mulu, Borneo, Malaysia	AB847560
76	*Leptobrachella sabahmontana*	BORNEENSIS 12632	Borneo, Malaysia	AB847551
77	*Leptobrachella dringi*	KUHE 55610	Camp 4 of Gunung Mulu, Malaysia	AB847553
78	*Leptobrachella picta*	UNIMAS 8705	Borneo, Malaysia	KJ831295
79	*Leptobrachella fritinniens*	KUHE 55371	Headquarters, Gunung Mulu, Malaysia	AB847557
80	*Leptobrachella sola*	KUHE 23261	Hala Bala, Thailand	LC202007
81	*Leptobrachella heteropus*	KUHE 15487	Larut, Peninsular, Malaysia	AB530453
82	*Leptobrachella kecil*	KUHE 52440	Malaysia	LC202004
83	*Leptobrachella kajangensis*	LSUHC 4439	Tioman, Malaysia	LC202002
84	*Leptobrachium tengchongense*	SYSa004604d	Yunnan Province, China	KX066880
85	*Leptobrachium huashen*	KIZ049025	Yunnan Province, China	KX811931
86	*Megophrys major*	AMS R 173870	Kon Tum, Vietnam	KY476333

### Molecular phylogenetic analyses

All five adult male specimens of the new species collected in this work were included in the molecular phylogenetic analyses (Table 1). For phylogenetic analyses, the corresponding gene sequences for all those related species for which comparable sequences were available were also downloaded from GenBank (Table 1). Corresponding sequences of one *Leptobrachium tengchongensis*, one *Leptobrachium huashen*, and one *Megophrys major* were downloaded (Table 1) and used as outgroups based on previous studies (Chen et al. 2018; Li et al. 2020a).

Total DNA was extracted using a standard phenol-chloroform extraction protocol (Sambrook et al. 1989). The mitochondrial 16S rRNA genes were amplified, and the primers P7 (5'-CGCCTGTTTACCAAAAACAT-3') and P8 (5'-CCGGTCTGAACTCAGATCACGT-3') were used following Simon et al. (1994). Gene fragments were amplified under the following conditions: an initial denaturing step at 95 °C for 4 min; 36 cycles of denaturing at 95 °C for 30 sec, annealing at 51 °C for 30 sec and extending at 72 °C for 70 sec. Sequencing was conducted using an ABI3730 automated DNA sequencer in Shanghai DNA BioTechnologies Co., Ltd. (Shanghai, China). New sequences were deposited in GenBank (for GenBank accession numbers see Table 1).

Sequences were assembled and aligned using the Clustalw module in BioEdit v. 7.0.9.0 (Hall 1999) with default settings. Alignments were checked by eye and revised manually if necessary. Phylogenetic analyses were conducted using maximum likelihood (ML) and Bayesian Inference (BI) methods, implemented in PhyML v. 3.0 (Guindon et al. 2010) and MrBayes v. 3.12 (Ronquist and Huelsenbeck 2003), respectively. We ran Jmodeltest v. 2.1.2 (Darriba et al. 2012) with Akaike and Bayesian information criteria on the alignment, resulting in the best-fitting nucleotide substitution models of GTR + I + G for the data. For the ML tree, branch supports were drawn from 10,000 nonparametric bootstrap replicates. In BI analyses, the parameters for each partition were unlinked, and branch lengths were allowed to vary proportionately across partitions. Two runs each with four Markov chains were simultaneously run for 50 million generations with sampling every 1,000 generations. The first 25% trees were removed as the “burn-in” stage followed by calculations of Bayesian posterior probabilities and the 50% majority-rule consensus of the post burn-in trees sampled at stationarity. Finally, genetic distance between *Leptobrachella* species based on uncorrected *p*-distance model was estimated on 16S gene using MEGA v. 6.06 (Tamura et al. 2013).

### Morphological comparisons

All five adult male specimens of the new species were measured (Table 2). The terminology and methods followed Fei et al. (2005), Mahony et al. (2011), and Wang et al. (2019). Measurements were made with a dial caliper to the nearest 0.1 mm (Watters et al. 2016) with digital calipers. Corresponding measurements of *L. bijie* and *L. chishuiensis* were retrieved from Wang et al. (2019) and Li et al. (2020a). Nineteen morphometric characters of adult specimens were measured:

**Table 2.** Measurements of adult males of *Leptobrachella jinshaensis* sp. nov. Units given in mm. See abbreviations for morphometric characters in Materials and methods section.

Voucher number	Sex	SVL	HDL	HDW	SL	IND	IOD	UEW	ED	TYD	LAL	LW	ML	THL	TW	TL	TFL	FL
CIBCS20200516001	male	31.1	11.4	10.1	4.9	3.4	3.1	2.8	3.9	2.5	15.4	2.6	8.4	15.0	4.9	15.3	21.4	14.4
CIBCS20200516002	male	31.2	10.8	10.4	4.6	3.2	3.2	2.7	3.9	2.8	13.7	2.1	7.7	15.2	3.2	15.6	19.3	13.0
CIBCS20200516003	male	29.7	10.0	10.1	4.6	3.2	3.4	3.0	4.2	2.5	14.4	2.2	7.2	14.0	3.6	15.1	19.5	13.0
CIBCS20200516004	male	31.1	10.3	10.0	4.5	2.8	3.7	2.9	4.3	2.6	15.2	2.4	8.2	14.6	3.5	15.1	21.4	14.2
CIBCS20200516005	male	30.9	11.3	10.4	4.6	3.5	4.0	3.2	3.7	3.2	14.1	2.2	8.2	14.1	3.6	14.5	21.2	14.2

**ED** eye diameter (distance from the anterior corner to the posterior corner of the eye);

**FL** foot length (distance from tarsus to the tip of the fourth toe);

**HDL** head length (distance from the tip of the snout to the articulation of jaw);

**HDW** head width (greatest width between the left and right articulations of jaw);

**HLL** hindlimb length (distance from tip of fourth toe to vent);

**IND** internasal distance (minimum distance between the inner margins of the external nares);

**IOD** interorbital distance (minimum distance between the inner edges of the upper eyelids);

**LAL** length of lower arm and hand (distance from the elbow to the distal end of the Finger IV);

**LW** lower arm width (maximum width of the lower arm);

**ML** manus length (distance from tip of third digit to proximal edge of inner palmar tubercle);

**SL** snout length (distance from the tip of the snout to the anterior corner of the eye);

**SVL** snout-vent length (distance from the tip of the snout to the posterior edge of the vent);

**TEY** tympanum-eye distance (distance from anterior edge of tympanum to posterior corner of eye);

**TFL** length of foot and tarsus (distance from the tibiotarsal articulation to the distal end of the toe IV);

**THL** thigh length (distance from vent to knee);

**TL** tibia length (distance from knee to tarsus);

**TW** maximal tibia width;

**TYD** maximal tympanum diameter;

**UEW** upper eyelid width (greatest width of the upper eyelid margins measured perpendicular to the anterior-posterior axis).

In order to reduce the impact of allometry, the correct value from the ratio of each character to SVL was calculated and then all of the data were log-transformed for the following morphometric analyses. Mann-Whitney *U* tests were conducted to test the significance of differences on morphometric characters between *Leptobrachella jinshaensis* sp. nov., *L. bijie* and *L. chishuiensis*. The significance level was set at 0.05. Furthermore, principal component analyses (PCA) were conducted to highlight whether the different species were separated in morphometric space. Due to only the measurements SVL, HDL, HDW, SL, IND, IOD, ED, TYD, TEY, LAL, ML, TL, HLL, and FL of male *L. bijie* being available from Wang et al. (2019), the morphometric analyses were conducted only based on these 14 morphometric characters for male group.

*Leptobrachella jinshaensis* sp. nov. was also compared with all other congeners of *Leptobrachella* based on morphological characters. Comparative morphological data were obtained from literatures (Table 3).

**Table 3.** References for morphological characters for congeners of the genus *Leptobrachella*.

No.	*Leptobrachella* species	References
1	*L. aerea* (Rowley, Stuart, Richards, Phimmachak & Sivongxay, 2010)	Rowley et al. 2010a
2	*L. alpina* (Fei, Ye & Li, 1990)	Fei et al. 1990
3	*L. applebyi* (Rowley & Cao, 2009)	Rowley and Cao 2009
4	*L. arayai* (Matsui, 1997)	Matsui 1997
5	*L. ardens* (Rowley, Tran, Le, Dau, Peloso, Nguyen, Hoang, Nguyen & Ziegler, 2016)	Rowley et al. 2016
6	*L. baluensis* (Smith, 1931)	Dring 1983; Eto et al. 2016, 2018
7	*L. bidoupensis* (Rowley, Le, Tran & Hoang, 2011)	Rowley et al. 2011
8	*L. bijie* (Wang, Li, Li, Chen & Wang, 2019)	Wang et al. 2019
9	*L. bondangensis* (Eto, Matsui, Hamidy, Munir & Iskandar, 2018)	Eto et al. 2018
10	*L. botsfordi* (Rowley, Dau & Nguyen, 2013)	Rowley et al. 2013
11	*L. bourreti* (Dubois, 1983)	Ohler et al. 2011
12	*L. brevicrus* (Dring, 1983)	Dring 1983; Eto et al. 2015
13	*L. chishuiensis* Li, Liu, Wei & Wang, 2020	Li et al. 2020a
14	*L. crocea* (Rowley, Hoang, Le, Dau & Cao, 2010)	Rowley et al. 2010b
15	*L. dringi* (Dubois, 1987)	Inger et al. 1995; Matsui and Dehling 2013
16	*L. eos* (Ohler, Wollenberg, Grosjean, Hendrix, Vences, Ziegler & Dubois, 2011)	Ohler et al. 2011
17	*L. feii* (Chen, Yuan & Che, 2020)	Chen et al. 2020
18	*L. firthi* (Rowley, Hoang, Dau, Le & Cao, 2012)	Rowley et al. 2012
19	*L. flaviglandulosa* (Chen, Yuan & Che, 2020)	Chen et al. 2020
20	*L. fritinniens* (Dehling & Matsui, 2013)	Dehling and Matsui 2013
21	*L. fuliginosa* (Matsui, 2006)	Matsui 2006
22	*L. fusca* (Eto, Matsui, Hamidy, Munir & Iskandar, 2018)	Eto et al. 2018
23	*L. gracilis* (Günther, 1872)	Günther 1872; Dehling 2012a
24	*L. hamidi* (Matsui, 1997)	Matsui 1997
25	*L. heteropus* (Boulenger, 1900)	Boulenger 1900
26	*L. isos* (Rowley, Stuart, Neang, Hoang, Dau, Nguyen & Emmett, 2015)	Rowley et al. 2015
27	*L. itiokai* Eto, Matsui & Nishikawa, 2016	Eto et al. 2016
28	*L. juliandringi* Eto, Matsui & Nishikawa, 2015	Eto et al. 2015
29	*L. kajangensis* (Grismer, Grismer & Youmans, 2004)	Grismer et al. 2004
30	*L. kalonensis* (Rowley, Tran, Le, Dau, Peloso, Nguyen, Hoang, Nguyen & Ziegler, 2016)	Rowley et al. 2016
31	*L. kecil* (Matsui, Belabut, Ahmad & Yong, 2009)	Matsui et al. 2009
32	*L. khasiorum* (Das, Tron, Rangad & Hooroo, 2010)	Das et al. 2010
33	*L. lateralis* (Anderson, 1871)	Anderson 1871; Humtsoe et al. 2008
34	*L. laui* (Sung, Yang & Wang, 2014)	Sung et al. 2014
35	*L. liui* (Fei & Ye, 1990)	Fei et al. 2009; Sung et al. 2014
36	*L. macrops* (Duong, Do, Ngo, Nguyen & Poyarkov, 2018)	Duong et al. 2018
37	*L. maculosa* (Rowley, Tran, Le, Dau, Peloso, Nguyen, Hoang, Nguyen & Ziegler, 2016)	Rowley et al. 2016
38	*L. mangshanensis* (Hou, Zhang, Hu, Li, Shi, Chen, Mo & Wang, 2018)	Hou et al. 2018
39	*L. maoershanensis* (Yuan, Sun, Chen, Rowley & Che, 2017)	Yuan et al. 2017
40	*L. marmorata* (Matsui, Zainudin & Nishikawa, 2014)	Matsui et al. 2014a
41	*L. maura* (Inger, Lakim, Biun & Yambun, 1997)	Inger et al. 1997
42	*L. melanoleuca* (Matsui, 2006)	Matsui 2006
43	*L. melica* (Rowley, Stuart, Neang & Emmett, 2010)	Rowley et al. 2010c
44	*L. minima* (Taylor, 1962)	Taylor 1962; Ohler et al. 2011
45	*L. mjobergi* (Smith, 1925)	Eto et al. 2015, 2018
46	*L. nahangensis* (Lathrop, Murphy, Orlov & Ho, 1998)	Lathrop et al. 1998
47	*L. namdongensis* (Hoang, Nguyen, Luu, Nguyen & Jiang, 2019)	Hoang et al. 2019
48	*L. natunae* (Günther, 1895)	Günther 1895
49	*L. neangi* (Stuart & Rowley, 2020)	Stuart and Rowley 2020
50	*L. niveimontis* (Chen, Yuan & Che, 2020)	Chen et al. 2020
51	*L. nokrekensis* (Mathew & Sen, 2010)	Mathew and Sen 2010
52	*L. nyx* (Ohler, Wollenberg, Grosjean, Hendrix, Vences, Ziegler & Dubois, 2011)	Ohler et al. 2011
53	*L. oshanensis* (Liu, 1950)	Liu 1950, 1961; This paper
54	*L. pallida* (Rowley, Tran, Le, Dau, Peloso, Nguyen, Hoang, Nguyen & Ziegler, 2016)	Rowley et al. 2016
55	*L. palmata* Inger & Stuebing, 1992	Inger and Stuebing 1992
56	*L. parva* Dring, 1983	Dring 1983
57	*L. pelodytoides* (Boulenger, 1893)	Boulenger 1893; Ohler et al. 2011
58	*L. petrops* (Rowley, Dau, Hoang, Le, Cutajar & Nguyen, 2017)	Rowley et al. 2017a
59	*L. picta* (Malkmus, 1992)	Malkmus 1992
60	*L. platycephala* (Dehling, 2012)	Dehling 2012b
61	*L. pluvialis* (Ohler, Marquis, Swan & Grosjean, 2000)	Ohler et al. 2000, 2011
62	*L. puhoatensis* (Rowley, Dau & Cao, 2017)	Rowley et al. 2017b
63	*L. purpuraventra* Wang, Li, Li, Chen & Wang, 2019	Wang et al. 2019
64	*L. purpurus* (Yang, Zeng & Wang, 2018)	Yang et al. 2018
65	*L. pyrrhops* (Poyarkov, Rowley, Gogoleva, Vassilieva, Galoyan & Orlov, 2015)	Poyarkov et al. 2015
66	*L. rowleyae* (Nguyen, Poyarkov, Le, Vo, Ninh, Duong, Murphy & Sang, 2018)	Nguyen et al. 2018
67	*L. sabahmontana* (Matsui, Nishikawa & Yambun, 2014)	Matsui et al. 2014b
68	*L. serasanae* Dring, 1983	Dring 1983
69	*L. shangsiensis* Chen, Liao, Zhou & Mo, 2019	Chen et al. 2019
70	*L. sola* (Matsui, 2006)	Matsui 2006
71	*L. suiyangensis* (Luo, Xiao, Gao & Zhou, 2020)	Luo et al. 2020
72	*L. sungi* (Lathrop, Murphy, Orlov & Ho, 1998)	Lathrop et al. 1998
73	*L. tadungensis* (Rowley, Tran, Le, Dau, Peloso, Nguyen, Hoang, Nguyen & Ziegler, 2016)	Rowley et al. 2016
74	*L. tamdil* (Sengupta, Sailo, Lalremsanga, Das & Das, 2010)	Sengupta et al. 2010
75	*L. tengchongensis* (Yang, Wang, Chen & Rao, 2016)	Yang et al. 2016
76	*L. tuberosa* (Inger, Orlov & Darevsky, 1999)	Inger et al. 1999
77	*L. ventripunctata* (Fei, Ye & Li, 1990)	Fei et al. 2009
78	*L. wuhuangmontis* Wang, Yang & Wang, 2018	Wang et al. 2018
79	*L. wulingensis* Qian, Xiao, Cao, Xiao & Yang, 2020	Qian et al. 2020
80	*L. yingjiangensis* (Yang, Zeng & Wang, 2018)	Yang et al. 2018
81	*L. yunkaiensis* Wang, Li, Lyu & Wang, 2018	Wang et al. 2018
82	*L. zhangyapingi* (Jiang, Yan, Suwannapoom, Chomdej & Che, 2013)	Jiang et al. 2013

### Bioacoustics data

The advertisement calls of *L. jinshaensis* sp. nov. were recorded from the holotype specimen CIBJS20200516004 in the field on 16 May 2020 in Lengshuihe Nature Reserve, Jinsha County, Guizhou Province, China. The advertisement call of *L. jinshaensis* sp. nov. was recorded in the stream at ambient air temperature of 20 °C and air humidity of 87%. A SONY PCM-D50 digital sound recorder was used to record within 20 cm of the calling individual. The sound files in wave format were resampled at 48 kHz with sampling depth 24 bits. Calls were recorded and examined as described by Wijayathilaka and Meegaskumbura (2016). Call recordings were visualised and edited with SoundRuler 0.9.6.0 (Gridi-Papp 2003–2007) and Raven Pro 1.5 software (Cornell Laboratory of Ornithology, Ithaca, NY, USA). Ambient temperature of the type locality was taken by a digital hygrothermograph. For comparison, bioacoustics data for the related species *L. bijie* and *L. chishuiensis* were obtained from Li et al. (2020a).

## Results

**Figure 2.**
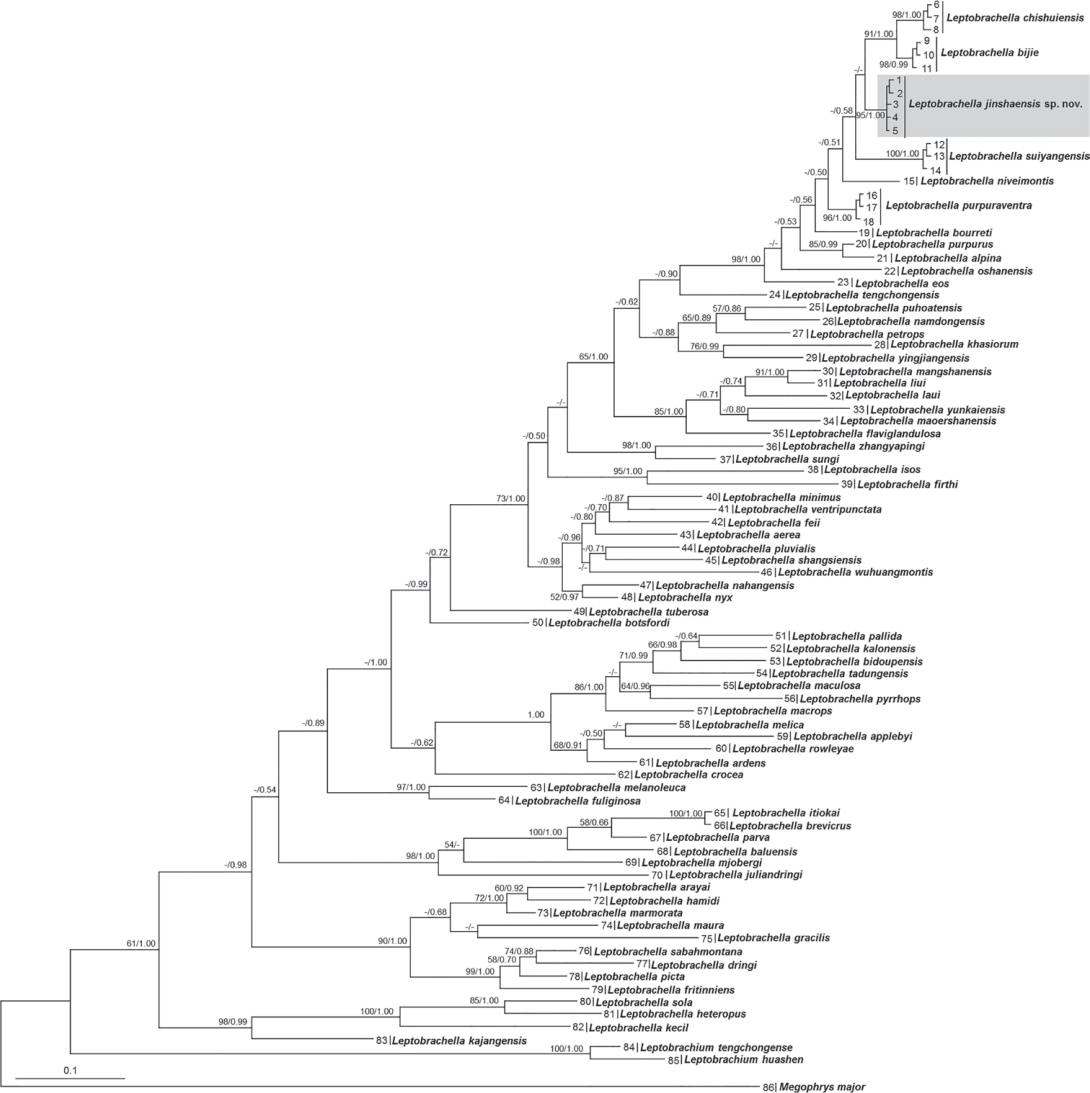
Bayesian Inference (BI) tree based on the mitochondrial 16S rRNA sequences. Bootstrap supports from Maximum Likelihood analyses/Bayesian posterior probabilities from BI analyses are labelled beside nodes. Information of samples 1–86 refer to Table 1.

Aligned sequence matrix of 16S rRNA gene contained 537 bps. ML and BI analyses resulted in essentially identical topologies (Fig. 2). All samples of *L. jinshaensis* sp. nov. were clustered into one independent clade nested into the *Leptobrachella* clade. The relationships between *L. jinshaensis* sp. nov. and its congeners are not resolved though it is likely sister to a clade in comprising of *L. bijie* and *L. chishuiensis* (Fig. 2). The smallest pairwise genetic divergence between *L. jinshaensis* sp. nov. and all other species of the genus *Leptobrachella* is 2.6% (vs. *L. niveimontis* or vs. *L. purpurus*), being at the same level with or higher than that between some pairs of substantial species, such as *L. bijie* vs. *L. chishuiensis* (2.1%), and *L. chishuiensis* vs. *L. alpina* (2.6%; Suppl. material 1: Table S1).

**Figure 3.**
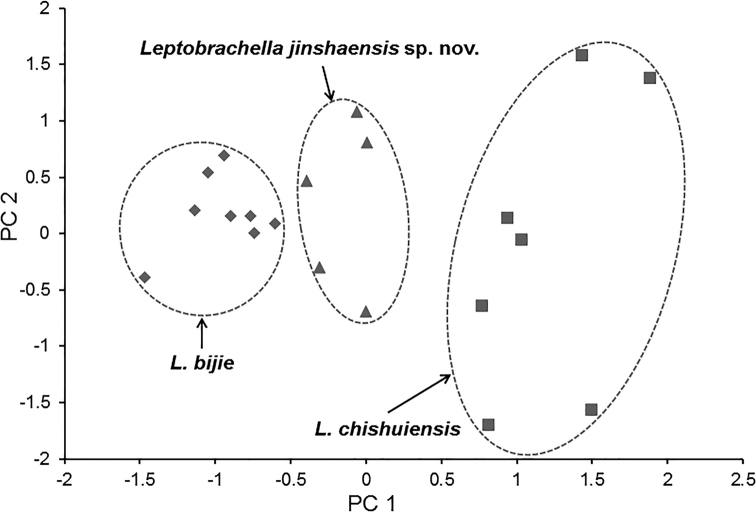
Plots of the first principal component (PC1) versus the second (PC2) for *Leptobrachella jinshaensis* sp. nov., *L. bijie*, and *L. chishuiensis* in males from a principal component analysis based on morphometric data.

For the male group, PCA extracted five principal component axes with eigenvalues greater than one, and the percentage of variance of the first five principal components are 37.7%, 15.7%, 13.0%, 9.0% and 8.1%, with percentage of cumulative is 83.5% (Suppl. material 2: Table S2). There were 14 morphological features with major contributions in the first five principal components, and these morphological features were distributed in the anterior, middle, and posterior parts of the body (Suppl. material 2: Table S2). The total variation of the first two principal components was 53.4% (Suppl. material 2: Table S2). On the PCA plot (PC1 vs. PC2), the first principal component axis could separate *L. jinshaensis* sp. nov. from *L. bijie* and *L. chishuiensis* (Fig. 3) mainly based on SVL, HDL, HDW, SL, ED, IND, TEY, and FL, and the second component axis mainly based on ML, FL, and LAL. Mann-Whitney *U* tests indicated that *L. jinshaensis* sp. nov. was significantly different from *L. bijie* on HDW, SL, IOD, TYD, TEY, LW, and FL, and from *L. chishuiensis* on SVL, TYD, and TL (*p*-values < 0.05; Table 4).

**Table 4.** Morphometric comparisons between *Leptobrachella jinshaensis* sp. nov. and its relatives. Units given in mm. See abbreviations for morphometric characters in Materials and methods section. *P*-value was resulted from Mann-Whitney *U* test. Significant level at 0.05. Abbreviations for species name: *LJ*, *Leptobrachella jinshaensis* sp. nov.; *LC*, *L. chishuiensis*; *LB*, *L. bijie.*

Character	*Leptobrachella jinshaensis* sp. nov.		*L. chishuiensis*	*L. bijie*			*P*-value	
	Male (n = 5)		Male (n = 7)	Male (n = 8)				
	Ranging	Mean ± SD	Ranging	Mean ± SD	Ranging	Mean ± SD	*LJ* vs. *LC*	*LJ* vs. *LB*
SVL	29.7–31.2	30.8 ± 0.6	30.8–33.4	32.1 ± 1.0	29.0–30.4	29.7 ± 0.6	0.088	0.019
HDL	10.0–11.4	10.7 ± 0.6	11.1–12.3	11.8 ± 0.4	10.0–10.6	10.2 ± 0.2	0.123	0.661
HDW	10.0–10.4	10.2 ± 0.2	10.6–11.9	11.4 ± 0.5	9.5–10.2	9.8 ± 0.3	0.012	0.463
SL	4.5–4.9	4.6 ± 0.1	4.8–5.8	5.2 ± 0.3	4.0–4.7	4.2 ± 0.2	0.019	0.057
IND	2.8–3.5	3.2 ± 0.3	3.5–3.8	3.7 ± 0.1	2.8–3.4	3.1 ± 0.2	0.062	0.464
IOD	3.1–4.0	3.5 ± 0.4	2.7–3.1	3.0 ± 0.2	2.8–3.4	3.1 ± 0.2	0.004	0.242
UEW	2.7–3.2	2.9 ± 0.2	3.0–3.3	3.2 ± 0.1	/	/	0.223	/
ED	3.7–4.3	4.0 ± 0.2	4.0–5.0	4.4 ± 0.4	3.6–4.1	3.8 ± 0.2	0.064	0.558
TYD	2.5–3.2	2.7 ± 0.3	2.0–2.6	2.3 ± 0.2	1.9–2.2	2.0 ± 0.1	0.019	0.003
TEY	0.9–1.4	1.0 ± 0.2	1.2–1.6	1.4 ± 0.2	0.9–1.1	1.0 ± 0.1	0.042	0.464
LAL	13.7–15.4	14.6 ± 0.7	14.7–17.0	15.6 ± 0.8	14.0–14.8	14.3 ± 0.3	0.570	0.661
LW	2.1–2.6	2.3 ± 0.2	2.6–3.2	3.0 ± 0.2	/	/	0.004	/
ML	7.2–8.4	7.9 ± 0.5	7.9–8.8	8.2 ± 0.39	7.4–8.3	7.8 ± 0.3	0.935	0.770
HLL	41.3–46.4	44.4 ± 2.0	43.3–49.7	49.7 ± 2.7	43.0–45.5	43.7 ± 0.8	0.291	0.464
THL	14.0–15.2	14.6 ± 0.5	13.7–17.1	15.1 ± 1.2	/	/	0.465	/
TW	3.2–4.9	3.8 ± 0.7	3.3–4.3	3.8 ± 0.4	/	/	0.935	/
TL	14.5–15.6	15.1 ± 0.4	14.9–16.8	15.6 ± 0.6	13.5–14.4	13. ± 0.3	0.685	0.008
TFL	19.3–21.4	20.6 ± 1.0	20.9–22.3	21.7 ± 0.6	/	/	0.962	/
FL	13.0–14.4	13.7 ± 0.7	14.4–15.9	15.1 ± 0.5	13.0–13.8	13.3 ± 0.2	0.019	0.558

In total, 109 advertisement calls of *Leptobrachella jinshaensis* sp. nov. were recorded in Lengshuihe Nature Reserve, Jinsa County, Guizhou Province, China on 16 May 2020 between 21:00–22:00. The call description is based on recordings of the holotype CIBJS20200516004 under a stone nearby a stream, and the ambient air temperature was 20 °C. The call characters of *L. jinshaensis* sp. nov. were demonstrated in the following section for describing it. There were some differences in sonograms and waveforms of calls between *L. jinshaensis* sp. nov., *L. bijie*, and *L. chishuiensis* (Suppl. material 3: Table S3). *Leptobranchella jinshaensis* sp. nov. has longer call interval (132.7 ± 8.6, *N* = 109) than *L. bijie* (101.9 ± 6.4, *N* = 33), and has lower dominant frequency (4525 ± 0.065 Hz) than *L. bijie* (4780.4 ± 76.5 Hz) and *L. chishuiensis* (6064–6284 H). Each call of *L. jinshaensis* sp. nov.has two kinds of notes, while each call of *L. chishuiensis* only has one kind of note.

### Leptobrachella jinshaensis sp. nov.

Taxon classificationAnimaliaAnuraMegophryidae

253F2320-A11B-512C-B8FF-90CC36AA0F70

http://zoobank.org/C2982600-D9EF-46C1-A539-CC1151444B18

**Figure 4.**
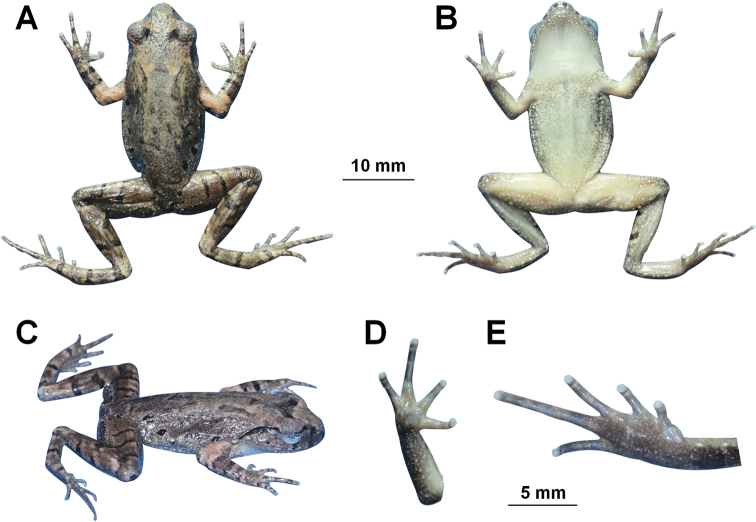
Photos of the holotype specimen CIBCS20200516004 of *Leptobrachella jinshaensis* sp. nov. **A** dorsal view **B** ventral view **C** lateral view **D** ventral view of hand **E** ventral view of foot.

**Figure 5.**
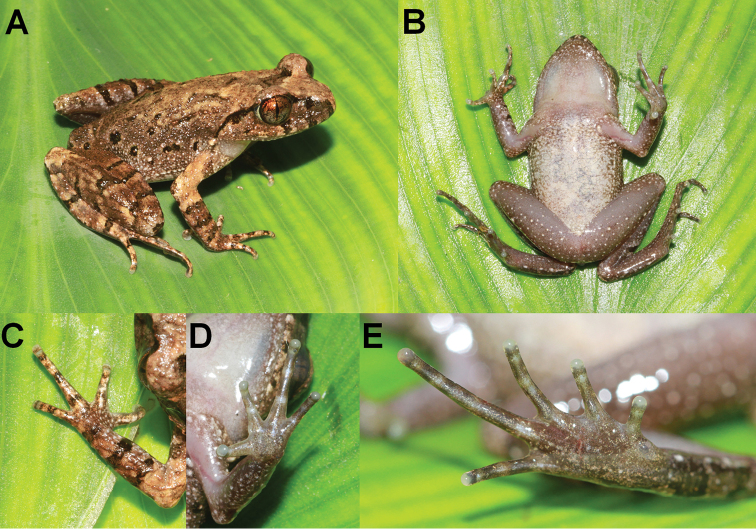
Photos of the holotype CIBCS20200516004 of *Leptobrachella jinshaensis* sp. nov. in life **A** dorsal view **B** ventral view **C** dorsal view of hand **D** ventral view of hand **E** ventral view of foot.

**Figure 6.**
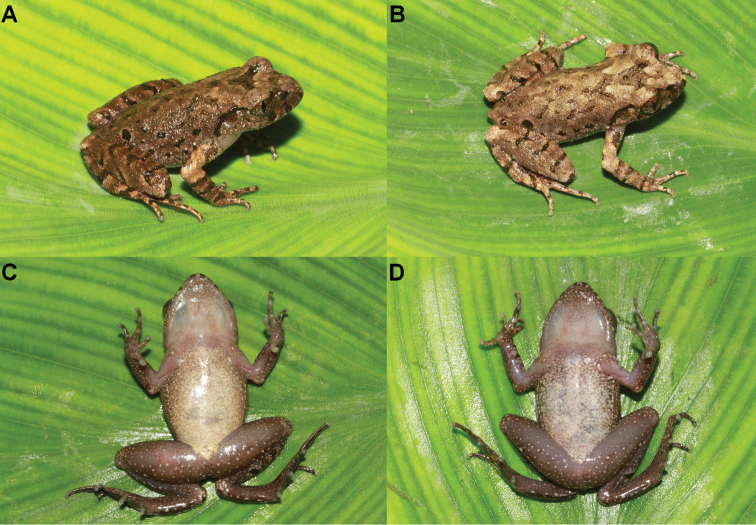
Colour variation in *Leptobrachella jinshaensis* sp. nov. **A** dorsal view of the male specimen CIBJS20200516002 **B** dorsal view of the male specimen CIBJS20200516005 **C** ventral view of the male specimen CIBJS20200516005 **D** ventral view of the male specimen CIBJS20200516003.

Figs 3 , 4 , 5 , 6 ; Tables 1 , 2 , 4 , Suppl. material 1 : Table S1, Suppl. material 2: Table S2

#### Holotype.

CIBJS20200516004, adult male (Figs 4, 5), collected from Lengshuihe Nature Reserve, Jinsha County (27.536944°N, 105.999166°E, ca. 770 m a. s. l.), Guizhou Province, China by Shi-Ze Li on 16 May 2020.

#### Paratypes.

Four adult males from the same place as holotype. Two adult males CIBJS20200516001 and CIBJS20200516002 collected by Shi-Ze LI, and two adult males CIBJS20200516003 and CIBJS20200516005 collected by Jing LIU, all of them were collected on 16 May 2020.

#### Diagnosis.

*Leptobrachella jinshaensis* sp. nov. is assigned to the genus *Leptobrachella* based on molecular phylogenetic analyses and the following morphological characters: medium size, rounded finger tips, the presence of an elevated inner palmar tubercle not continuous to the thumb, the presence of macroglands on body (including supra-axillary, pectoral, and femoral glands), vomerine teeth absent, tubercles on eyelids, and the anterior tip of snout with a vertical white bar.

*Leptobrachella jinshaensis* sp. nov. can be distinguished from its congeners by a combination of the following characters: body of medium size (SVL 29.7–31.2 mm in five adult males); dorsal skin shagreened, some of the granules forming longitudinal short skin ridges; tympanum distinctly discernible, slightly concave; supra-axillary, femoral, pectoral, and ventrolateral glands distinctly visible; absence of webbing and lateral fringes on fingers; toes with narrow lateral fringes and without webbing; heels overlapping when thighs positioned at right angles to the body; tibia-tarsal articulation reaching the middle eye when leg stretched forward.

#### Description of holotype

**(Figs 4, 5).** Adult male. SVL in 31.1 mm. ***Head*** length slightly longer than head width (HDL/HDW 1.02); snout slightly protruding, projecting slightly beyond margin of the lower jaw; nostril closer to snout than eye; canthus rostralis gently rounded; loreal region slightly concave; interorbital space ﬂat, interorbital distance slightly longer than internarial distance; pineal ocellus absent; vertical pupil; eye diameter slightly shorter than snout length; tympanum distinct, rounded, and slightly concave, diameter smaller than that of the eye (TMP/ED 0.61); upper margin of tympanum in contact with supratympanic ridge; vomerine teeth absent; tongue notched behind; supratympanic ridge distinct, extending from posterior corner of eye to supra-axillary gland.

***Forelimbs*** slender, 48.9% of snout-vent length; tips of fingers rounded, slightly swollen; relative finger lengths I < II <= IV < III; absence of webbing; nuptial pad and subarticular tubercles absent; inner palmar tubercle large, rounded separated from the smaller, round outer palmar tubercle.

***Hindlimbs*** slender, tibia slightly longer than thigh length and 48.4% of snout-vent length; heels overlapping when thighs are positioned at right angles to the body, tibiotarsal articulation reaching middle eye when leg stretched forward; relative toe lengths I < II < V < III < IV; tips of toes round, slightly dilated; subarticular tubercle at the articulations of the toes absent; toes without webbing; lateral fringes narrow on all toes; inner metatarsal tubercle present, large, oval, outer metatarsal tubercle absent.

Dorsal surface shagreened and granular, some of the granules forming short longitudinal folds dorsally on the flank; ventral skin smooth; dense tiny granules present on ventral surface of thigh and tibia; pectoral gland and femoral gland white, oval, distinctly visible. Ventrolateral gland distinctly visible and forming an incomplete line.

#### Colouration of holotype in life.

Dorsum brown, with small, distinct darker brown markings and spots, and irregularly dispersed light orange speckles. A dark brown inverted triangular pattern between anterior corners of eyes. Tympanum brown, a dark brown bar above tympanum, and a dark brown bar under the eye, distinct black supratympanic line present; transverse dark brown bars on dorsal surface of limbs; distinct dark brown blotches on ﬂanks from groin to axilla, longitudinally in two rows; elbow and upper arms with dark bars and distinct coppery orange colouration; fingers and toes with distinct dark bars. Ventral surface of throat cream white, chest, and belly cream yellow with purple speckling, and on ﬂanks presence of distinct nebulous greyish speckling; ventral surface of limbs grey purple. Supra-axillary gland, femoral, pectoral, and ventrolateral glands white (Fig. 5).

#### Colouration of holotype in preservation.

Dorsum of body and limbs fade to brown copper; transverse bars on limbs become more distinct. Ventral surface of body and limbs fade to cream white. Supra-axillary, femoral, and pectoral glands fade to creamy yellow (Fig. 4).

#### Variation.

Measurements of adult specimens were presented in Tables 2 and 4. All specimens were similar but some individuals different from the holotype in colour pattern. In CIBJS20200516002, the tympana are dark brown (Fig. 6A); in CIBJS20200516005, the dorsum is olive grey (Fig. 6B) and the pectoral glands on the left side not obviously (Fig. 6D); in CIBJS20200516003 ventrolateral glands scattered and unlined (Fig. 6C).

#### Advertisement call.

**Figure 7.**
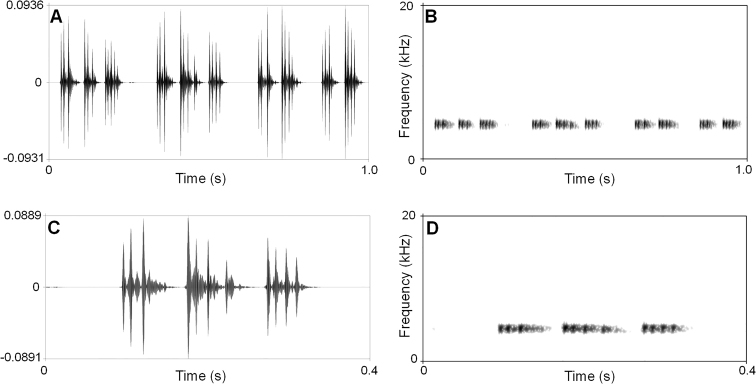
Advertisement calls of the holotype CIBCS20200516004 of *Leptobrachella jinshaensis* sp. nov. **A** waveform showing one second contains 4 calls **B** sonogram showing one second contains 4 calls **C** waveform showing 0.4 second contains a call **D** sonogram showing 0.4 second contains a call.

In total, 109 advertisement calls of *Leptobrachella jinshaensis* sp. nov. were recorded in Lengshuihe Nature Reserve, Jinsa County, Guizhou Province, China on 16 May 2020 between 21:00–22:00. The call description is based on recordings of the holotype CIBJS20200516004 under a stone nearby a stream, and the ambient air temperature was 20 °C. The sonograms and waveforms of the new species are shown in Fig. 7 and Suppl. material 2: Table S2. The call has two kinds of notes, and each call contains two or three notes (mean 2.12 ± 0.33, n = 109). Call duration was 117–156 ms (mean 132.7 ± 8.6, n = 109). Call interval was 62–106 ms (mean 84.3 ± 10.4, n = 108), and each consists of two types of note. The first type of note is the start note in each call and beginning with lowest energy pulses, then increasing to the peak; in the second type, the amplitude begins with highest pulses and then decreasing towards the end of each note. The duration of first type of note with 35–71 ms (mean 48.77 ± 7.90, n = 109), the duration of the second type of note with 39–78 ms (mean 52.93 ± 8.85, n = 122), the duration between notes 18–40 ms (mean 23 ± 5.68, n = 122). The dominant frequency of calls is 4500–4688 Hz (mean 4525 ± 0.065 Hz).

#### Secondary sexual characteristics.

Adult males with a comparatively large single subgular vocal sac and nuptial pads and spines absent.

#### Comparisons.

Measurements were given in mm. In male, by body size moderate in male (SVL 29.7–31.2, n = 5), *Leptobrachella jinshaensis* sp. nov. is larger than *L. aerea* (25.1–28.9), *L. alpina* (24.0–26.4), *L. applebyi* (19.6–22.3), *L. ardens* (21.3–24.7), *L. baluensis* (14.9–15.9), *L. bidoupensis* (18.5–25.4), *L. bondangensis* (17.8), *L. brevicrus* (17.1–17.8), *L. crocea* (22.2–27.3), *L. feii* (21.5–22.8), *L. flaviglandulosa* (23.0–27.0), *L. fusca* (16.3), *L. isos* (23.7–27.9), *L. itiokai* (15.2–16.7), *L. juliandringi* (17.0–17.2), *L. khasiorum* (24.5–27.3), *L. laui* (24.8–26.7), *L. maculosa* (24.2–26.6), *L. mangshanensis* (22.22–27.76), *L. maura* (26.1), *L. melica* (19.5–22.8), *L. mjobergi* (15.7–19.0), *L. natunae* (17.6), *L. niveimontis* (22.5–23.6), *L. parva* (15.0–16.9), *L. palmata* (14.4–16.8), *L. pallida* (24.5–27.7), *L. petrops* (23.6–27.6), *L. pluvialis* (21.3–22.3), *L. purpurus* (25.0–27.5), *L. rowleyae* (23.4–25.4), *L. serasanae* (16.9), *L. tengchongensis* (23.9–26.0), *L. ventripunctata* (25.5–28.0), and *L. yingjiangensis* (25.7–27.6); and smaller than *L. eos* (33.1–34.7), *L. gracilis* (34.3–39.0), *L. marmorata* (32.3–38.0), *L. nahangensis* (40.8), *L. platycephala* (35.1), *L. sungi* (48.3–52.7), *L. tamdil* (32.0), and *L. zhangyapingi* (45.8–52.5).

By the presence of supra-axillary and ventrolateral glands, *Leptobrachella jinshaensis* sp. nov. can be easily distinguished from *L. arayai*, *L. dringi*, *L. fritinniens*, *L. gracilis*, *L. hamidi*, *L. heteropus*, *L. kajangensis*, *L. kecil*, *L. marmorata*, *L. melanoleuca*, *L. maura*, *L. picta*, *L. platycephala*, *L. sabahmontana*, and *L. sola* (vs. lacking supra-axillary and ventrolateral glands in the latter).

By tympanum distinctly visible, *Leptobrachella jinshaensis* sp. nov. differs from *L. crocea* and *L. tuberosa* (vs. invisible in the latter).

By having black spots on ﬂanks, *Leptobrachella jinshaensis* sp. nov. differs from *L. aerea*, *L. botsfordi*, *L. firthi*, *L. crocea*, *L. isos*, *L. pallida*, *L. petrops*, and *L. tuberosa* (vs. lacking in the latter).

By toes without webbing, *Leptobrachella jinshaensis* sp. nov. differs from *L. aerea*, *L. alpina*, *L. applebyi*, *L. bidoupensis*, *L. bijie*, *L. botsfordi*, *L. bourreti*, *L. chishuiensis*, *L. crocea*, *L. eos*, *L. feii*, *L. firthi*, *L. fuliginosa*, *L. isos*, *L. khasiorum*, *L. lateralis*, *L. laui*, *L. liui*, *L. macrops*, *L. mangshanensis*, *L. maoershanensis*, *L. marmorata*, *L. melica*, *L. minima*, *L. nahangensis*, *L namdongensis*, *L. niveimontis*, *L. nokrekensis*, *L. nyx*, *L. pluvialis*, *L. pluvialis*, *L. puhoatensis*, *L. purpurus*, *L. purpuraventra*, *L. pyrrhops*, *L. sabahmontaus*, *L. shangsiensis*, *L. suiyangensis*, *L. tengchongensis*, *L. tuberosa*, *L. ventripunctata*, *L. wuhuangmontis*, *L. yingjiangensis*, *L. yunkaiensis*, and *L. zhangyapingi* (vs. webbing rudimentary in the latter); and differs from *L. flaviglandulosa* and *L. pelodytoides* (vs. webbing present in the latter).

By toes with narrow lateral fringes, *Leptobrachella jinshaensis* sp. nov. differs from *L. aerea*, *L alpina*, *L. firthi*, *L. laui*, *L. liui*, *L. khasiorum*, and *L. yunkaiensis* (vs. wide in the latter); and differs from *L. kalonensis*, *L. macrops*, *L. minima*, *L. marmorata*, *L. namdongensis*, *L. nyx*, *L. oshanensis*, *L. pyrrhops*, *L. rowleyae*, and *L. tuberosa* (vs. lacking in the latter).

By dorsal surface shagreened and granular, lacking enlarge tubercles or warts, *Leptobrachella jinshaensis* sp. nov. differs from *L. applebyi*, *L. bidoupensis*, *L. kalonensis*, *L. melica*, *L. minima*, *L. nahangensis*, *L. shangsiensis*, and *L. tadungensis* (all of which have the dorsum smooth), and *L. bourreti* (dorsum smooth with small warts), *L. fuliginosa* (dorsum smooth with fine tubercles), *L. liui* (dorsum with round tubercles), *L. macrops* (dorsum roughly granular with large tubercles), *L. maoershanensis* (dorsum shagreened with tubercles), *L. minima* (dorsum smooth), *L. nyx* (dorsum with round tubercles), *L. nokrekensis* (dorsum tubercles and longitudinal folds), *L. pelodytoides* (dorsum with small, smooth warts), *L. tamdil* (dorsum weakly tuberculate, with low, oval tubercles), *L. tuberosa* (dorsum very tuberculate), *L. yunkaiensis* (dorsum with raised warts), and *L. wuhuangmontis* (dorsum rough with conical tubercles).

By having higher dominant frequency (4.5–4.7 kHz, 20 °C), *Leptobrachella jinshaensis* sp. nov. differs from *L. applebyi* (3.9–4.3 kHz, 21.5 °C), *L. ardens* (3.1–3.4 kHz, 23.6 °C), *L. bidoupensis* (1.9–2.3 kHz, 19.9 °C), *L. botsfordi* (2.6–3.2 kHz, 14 °C), *L. crocea* (2.6–3.0 kHz, 21.6–25.1 °C), *L. fuliginosa* (2.3–2.4 kHz,19.3–19.6 °C), *L. heteropus* (2.8 kHz, 21 °C), *L. maculosa* (2.7 kHz, 23.3–24.1 °C), *L. melanoleuca* (3.1–3.3 kHz, 23.9 °C), *L. melica* (2.9–3.8 kHz, 26.1 °C), *L. pallida* (2.4–2.7 kHz, 18.9 °C), *L. pyrrhops* (1.9–22 kHz, 25 °C), *L. rowleyae* (2.6–3.0 kHz, 21.6–25.1 °C), *L. sola* (3.1–3.2 kHz, 24.2–24.3 °C), L. tadungensis (2.6–3.1 kHz, 12.9–22.3 °C) and *L. tuberosa* (2.6–2.8 kHz, 22.5–24.5 °C). The call of the new species appears to have lower frequency compared to the calls attributed to *L. aerea* (6.2–6.4 kHz, 22.4 °C), *L. isos* (7.83–8.55 kHz, 26.4 °C), *L. marmorata* (6.0–6.2 kHz, 22.8 °C), *L. pelodytoides* (6.4–6.6 kHz, 22.7 °C), *L. ventripunctata* (6.1–6.4 kHz, 15 °C) and *L. yingjiangensis* (5.7–5.9 kHz, 19 °C).

By call duration 117–156 ms, *Leptobrachella jinshaensis* sp. nov. differs from *L. aerea* (16–28 ms), *L. bidoupensis* (308–400), *L. botsfordi* (239–303 ms), *L. firthi* (18–24 ms), *L. fuliginosa* (51–80 ms), *L. isos* (31–38 ms), *L. maculosa* (889–907 ms), *L. marmorata* (1900–6700 ms), *L. melanoleuca* (40–63 ms) , *L. pallida* (627–729 ms), *L. petrops* (44–57 ms), *L. puhoatensis* 6–14 ms, *L. shangsiensis* (64–69 ms), *L. tadungensis* (248–353 ms) and *L. yingjiangensis* (28–42 ms).

Seven species (*L. liui*, *L. oshanensis*, *L. purpuraventra*, *L. bijie*, *L. suiyangensis*, *L. chishuiensis*, and *L. ventripunctata*) of the genus occur in Guizhou Province, China (Fei et al. 2012; Wang et al. 2019; Luo et al. 2020; Li et al. 2020a). The new species differs from *L. liui* by having narrow lateral fringes on toes (vs. wide in the latter), dorsal surface shagreened with small granules, and lacking enlarge tubercles or warts (vs. dorsum with round tubercles in the latter); differs from *L. oshanensis* by having narrow lateral fringes on toes (vs. lacking in the latter); differs from *L. purpuraventra* and *L. suiyangensis* by heels overlapping when thighs are positioned at right angles to the body (vs. just meeting in the latter); differs from *L. purpuraventra* by tibia-tarsal articulation reaches the middle eye when leg stretched forward (vs. only reaches the level between tympanum to eye in the latter).

In mitochondrial DNA trees, *Leptobrachella jinshaensis* sp. nov. clustered as an independent clade and appears to be sister to a clade in comprising of *L. bijie* and *L. chishuiensis*. The latter two species also occur near the type locality of the new species. The new species differs from *L. bijie* by the following characters: webbing on toes absent (vs. webbing rudimentary in the latter), heels overlapping when thighs are positioned at right angles to the body (vs. just meeting in the latter), having longer call interval (132.7 ± 8.6, *N* = 109 in the new species vs.101.9 ± 6.4, *N* = 33 in the latter), having lower dominant frequency of 4525 ± 0.065 Hz vs. 4780.4 ± 76.5 Hz in the latter, having significantly higher value of SVL in males, and having significantly higher value of TYD and TL to SVL in males. *Leptobrachella jinshaensis* sp. nov. differs from *L. chishuiensis* by webbing on toes absent (vs. webbing rudimentary in the latter), tibia-tarsal articulation reaches the middle of eye when leg stretched forward (vs. reaches the tympanum or the level between tympanum to eye in the latter), the lower dominant frequency of calls 4500–4688 Hz (mean 4525 ± 0.065, 20 °C) vs. 6064–6284 Hz (6140.15 ± 69.35, 20 °C) in the latter, each call with two kinds of notes vs. only one kind of note in the latter, and having significantly higher value of HDW, SL, IOD, TYD, TEY and FL to SVL in males (all *p*-values < 0.05; Table 4).

#### Ecology.

**Figure 8.**
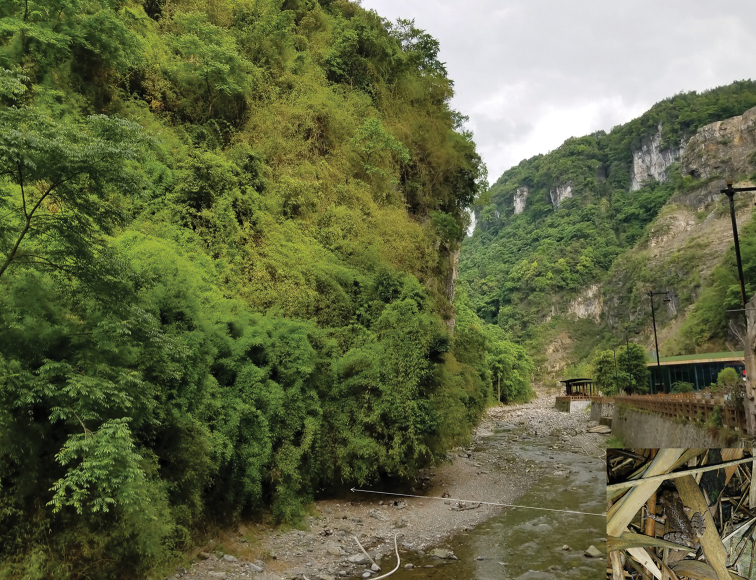
Habitats of *Leptobrachella jinshaensis* sp. nov. in the type locality Lengshuihe Nature Reserve, Jinsha County, Guizhou Province, China. Forest and a mountain stream in the type locality (*insert* holotype CIBCS20200516004 in life in the field).

*Leptobrachella jinshaensis* sp. nov. is known from the type locality, Lengshuihe Nature Reserve, Jinsha County, Guizhou Province, China. Specimens of the new species are frequently found from stream covered with reeds, and under the rocks (Fig. 8).

#### Etymology.

The specific name *jinshaensis* refers to the distribution of this species, Jinsha County, Guizhou Province, China. We suggest its English common name “Jinsha leaf litter toads” and Chinese name “Jin Sha Zhang Tu Chan (金沙掌突蟾)”.

## Discussion

Molecular phylogenetic analyses, detailed morphological comparisons, and advertisement call data all supported the new species distinctly separated from its congeners especially the superficially-morphological-similar species, *L. bijie* and *L. chishuiensis*. Although the relationships between the new species and other closely related species were not resolved, the new species appears to be phylogenetically closer to *L. bijie* and *L. chishuiensis*, corresponding to their high similarity on morphology. However, the new species appears to have lower dominant frequency on calling than the two closely related species. Moreover, they could be separated by morphometric analyses on contributions of some characters, for example, on PC1 of PCA, several characters of head, SVL and FL, which might be associated the calling behaviours, breeding behaviours, and jumping behaviours. We need future work to detect the function of the characters of these species to explore the ecological differences between them.

The large-scale molecular phylogenetic analyses in Chen et al. (2018) revealed many cryptic species in the genus *Leptobrachella* but did not included samples of *Leptobrachella jinshaensis* sp. nov. Similarly, this large phylogenetic framework likely included a few population samples in Guizhou Province, China. However, the phylogenetic framework indicated that Guizhou Province might be the biogeographical zone of transition for western-to-eastern or southwestern-to-northeastern clades (Chen et al. 2018). The findings of series of new species (*Leptobrachella jinshaensis* sp. nov., *L. chishuiensis*, *L. suiyangensis*, *L. bijie*, and *L. purpuraventra*) obviously supply important supplemental materials for detecting detailed evolutionary and biogeographical models of the genus. Moreover, the findings of the new species also indicated a high degree of localised diversification and micro-endemism for the species in the genus *Leptobrachella* because in Guizhou Province, China, the five recent-described *Leptobrachella* species are just known only from their type localities or nearby areas. In addition, in recent years, large number of discoveries have been made from Guizhou, dramatically raising the number of frog species known from the region (Zhang et al. 2017; Li et al. 2018a, b, 2019a, b, 2020a, b, c; Lyu et al. 2019; Wang et al. 2019; Luo et al. 2020; Su et al. 2020; Xu et al. 2020; Wei et al. 2020). This further indicated that more investigations should be conducted in Guizhou Province to define more precisely distribution area of the new species and detect more cryptic species especially in the poorly-investigated areas.

## Supplementary Material

XML Treatment for Leptobrachella jinshaensis

## Supplementary materials

Supplementary material 1 Table S1. Uncorrected *p*-distance between *Leptobrachella* species on the 16S rRNA gene Data type molecular data https://binary.pensoft.net/file/515461This dataset is made available under the Open Database License (http://opendatacommons.org/licenses/odbl/1.0/). The Open Database License (ODbL) is a license agreement intended to allow users to freely share, modify, and use this Dataset while maintaining this same freedom for others, provided that the original source and author(s) are credited.Bin Wang

Supplementary material 2 Table S2. Variable loadings for principal components with Eigenvalue greater than 1, from morphometric characters corrected by SVL Data type species data https://binary.pensoft.net/file/515462This dataset is made available under the Open Database License (http://opendatacommons.org/licenses/odbl/1.0/). The Open Database License (ODbL) is a license agreement intended to allow users to freely share, modify, and use this Dataset while maintaining this same freedom for others, provided that the original source and author(s) are credited.Yan-Lin Cheng, Sheng-Chao Shi, Jiaqi Li, Jing Liu, Shi-Ze Li, Bin Wang

Supplementary material 3 Table S3. Advertisement call comparisons between *Leptobrachella jinshaensis* sp. nov. and its congeners Data type statistical data https://binary.pensoft.net/file/515463This dataset is made available under the Open Database License (http://opendatacommons.org/licenses/odbl/1.0/). The Open Database License (ODbL) is a license agreement intended to allow users to freely share, modify, and use this Dataset while maintaining this same freedom for others, provided that the original source and author(s) are credited.Yan-Lin Cheng, Sheng-Chao Shi, Jiaqi Li, Jing Liu, Shi-Ze Li, Bin Wang
